# Differences in Neural-Immune Gene Expression Response in Rat Spinal Dorsal Horn Correlates with Variations in Electroacupuncture Analgesia

**DOI:** 10.1371/journal.pone.0042331

**Published:** 2012-08-03

**Authors:** Ke Wang, Rong Zhang, Xiaohui Xiang, Fei He, Libo Lin, Xingjie Ping, Lei Yu, Jisheng Han, Guoping Zhao, Qinghua Zhang, Cailian Cui

**Affiliations:** 1 Shanghai-MOST Key Laboratory of Health and Disease Genomics, Chinese National Human Genome Center at Shanghai and National Engineering Research Center for Biochip at Shanghai, Shanghai, China; 2 Neuroscience Research Institute; Department of Neurobiology, Peking University Health Science Center; Key Laboratory of Neuroscience of the Ministry of Education and the Ministry of Public Health; Peking University, Beijing, China; 3 Department of Genetics and Center of Alcohol Studies, Piscataway, New Jersey, United States of America; 4 Department of Microbiology and Li Ka Shing Institute of Health Sciences, The Chinese University of Hong Kong, Prince of Wales Hospital, Shatin, New Territories, Hong Kong Special Administrative Region, China; University of Pecs Medical School, Hungary

## Abstract

**Background:**

Electroacupuncture (EA) has been widely used to alleviate diverse pains. Accumulated clinical experiences and experimental observations indicated that significant differences exist in sensitivity to EA analgesia for individuals of patients and model animals. However, the molecular mechanism accounting for this difference remains obscure.

**Methodology/Principal Findings:**

We classified model male rats into high-responder (HR; TFL changes >150) and non-responder (NR; TFL changes ≤0) groups based on changes of their pain threshold detected by tail-flick latency (TFL) before and after 2 Hz or 100 Hz EA treatment. Gene expression analysis of spinal dorsal horn (DH) revealed divergent expression in HR and NR after 2 Hz/100 Hz EA. The expression of the neurotransmitter system related genes was significantly highly regulated in the HR animals while the proinflammation cytokines related genes were up-regulated more significantly in NR than that in HR after 2 Hz and 100 Hz EA stimulation, especially in the case of 2 Hz stimulation.

**Conclusions/Significance:**

Our results suggested that differential regulation and coordination of neural-immune related genes might play an important role for individual variations in analgesic effects responding to EA in DH. It also provided new candidate genes related to EA responsiveness for future investigation.

## Introduction

Acupuncture has been used in China and other Asian countries for more than 2500 years and in western countries for decades [1]. Instead of traditional acupuncture manipulations, electroacupuncture (EA) has been used with electric pulses delivered to acupuncture needles to alleviate pains of diverse etiology [2], [3], [4]. Although EA analgesia was considered generally effective in practice, significant individual variations in analgesic effects were well documented in both animals and humans [5], [6]. According to the magnitude of the analgesic response to EA, an individual can be categorized into high-responders (HR) and non-responders (NR) [6], [7]. In some previous animal studies, rats showing a statistically significant increase in tail flick latency (TFL) by EA stimulation (*P*<0.01) were classified as HR, and other subjects were classified as NR [8], [9], [10]. In other studies, rats showing an increase more than 30% or 60% in TFL in response to EA stimulation were classified as HR, while rats showing less than a 20% or 30% increase in TFL in response to EA were classified as NR [6], [11]. Because there were different criteria for definition of the HR and NR, the percentage for HR and NR in previous reports were 30.0%∼61.1% and 30.0%∼46.2%, respectively [6], [8], [9], [10], [11], [12]. This phenomenon leaves an obstacle to clinical pain management by EA treatment, especially to the application of acupuncture compound anesthesia in clinical surgical operation.

Further research has been done to demonstrate a dynamic balance process between nociceptive and antinociceptive substances in CNS involved in acupuncture analgesia as well as individual variations in acupuncture analgesia [4]. Analgesic effect of EA has been shown to be due to the release of endogenous opioid peptides and activation of the descending inhibitory pathways in the central nervous system (CNS) [13]. The different neuropeptides are released in response to EA with different frequencies. For instance, low-frequency (2 Hz) EA accelerates the release of enkephalin, β-endorphin and endomorphin. In contrast, EA with high-frequency (100 Hz) selectively increases dynorphin release. Furthermore, many other neurotransmitters and/or receptors in CNS also play a role mediating EA analgesia [14], [15]. These results of these studies suggest that the effect of EA is mediated by many substances and pathways in CNS. In addition, previous studies have demonstrated that the endogenous antiopioid peptide cholecystokinin (Cck) release and the density of the postsynaptic CCK receptors (Cck-a and -b) in the CNS are closely associated with individual sensitivity to EA [9], [10], [16]. Though Cck mechanisms have been well understood for individual differences in response to EA stimulation, the molecular mechanisms underlying individual differences in response to EA analgesia are largely unknown.

**Figure 1:**
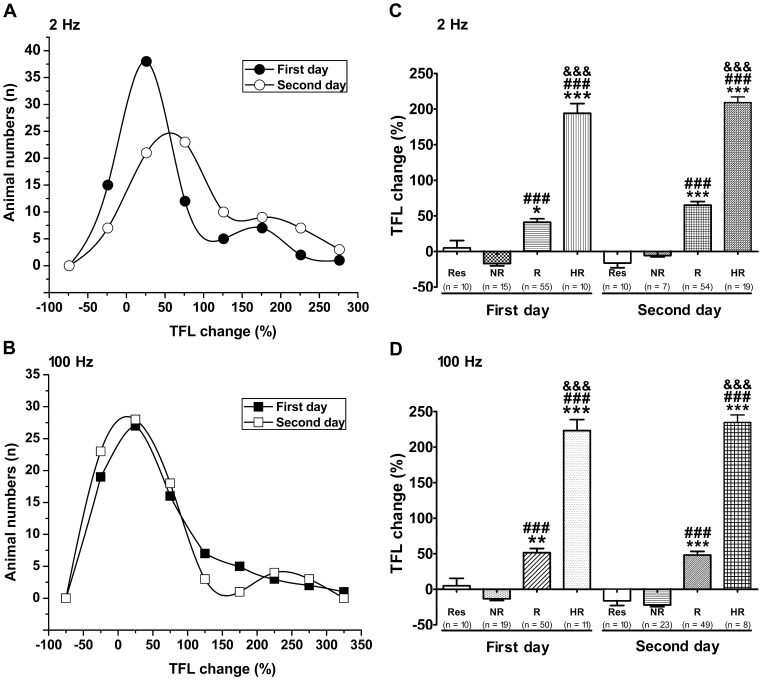
Analgesic effect induced by 2 Hz and 100 Hz EA in rats. The analgesic effects of EA on acute thermal pain were quantified using the TFL test, and they are expressed as percent changes from basal TFL. Either 2 Hz (A) or 100 Hz (B) EA, rats showed individual differences in sensitivity of EA analgesia for two consecutive days. A majority of rats showed sensitivity to EA analgesia (TFL change >0) and a small part appeared in contrary way (TFL change ≤0). Plan C and D showed comparison of the EA analgesic effects among the high-responder (HR), responder (R), and non-responder (NR) or restraint (Res) group in two consecutive days. Data are represented as mean ± SEM. One way ANOVA and Bonferroni’s Multiple Comparison Test was used to analysis TFL change (%). *P<0.05, **P<0.01, ***P<0.001 vs. restraint group; ^###^ *P*<0.001, vs. compared with the corresponding NR group; ^&&&^ *P*<0.001, compared with the corresponding R groups.

Gene expression in the CNS is the first measurable indicator of the interaction between genome and stimulation response [17]. Gene expression profiling of particular regions of CNS is used to decipher the molecular bases of changes in special function and behavior in response to environmental changes in modern neurobiology. Thus, we hypothesized that the differences in gene expression response may result in individual variations in EA analgesia. The spinal dorsal horn (DH) is an important direct responding region involved in EA analgesia [18], [19], [20], and an important relay site in the transmission of nociceptive information from the periphery to the brain, and nociception modulation by descending influences from the brainstem to local mechanisms [21], [22]. Aiming at elucidating the molecular basis of complex traits of behaviors in response to various kinds of stimulations, Sprage-Dawley (SD) rats were commonly used to model the human genetic complexity of individual variations in response to EA stimulation [23], [24]. In the present study, in order to explore the molecular mechanisms underlying the individual variation in analgesic response to 2 Hz and 100 Hz stimulation, we studied and compared the gene expression profiles in the DH in HR versus NR rats.

## Materials and Methods

### Animals

All experiments were performed on male SD rats, obtained from the Experimental Animal Center, Peking University, weighing 200–220 g at the beginning of the experiment. Animals were housed in a 12 h light/dark cycle with food and water *ad libitum*. The room temperature was maintained at 22±1°C and relative humidity at 45–50%. Rats were handled daily during the first three days after arrival. All experimental procedures were approved by the Animal Care and Use Committee of Peking University Health Science Centre.

**Figure 2:**
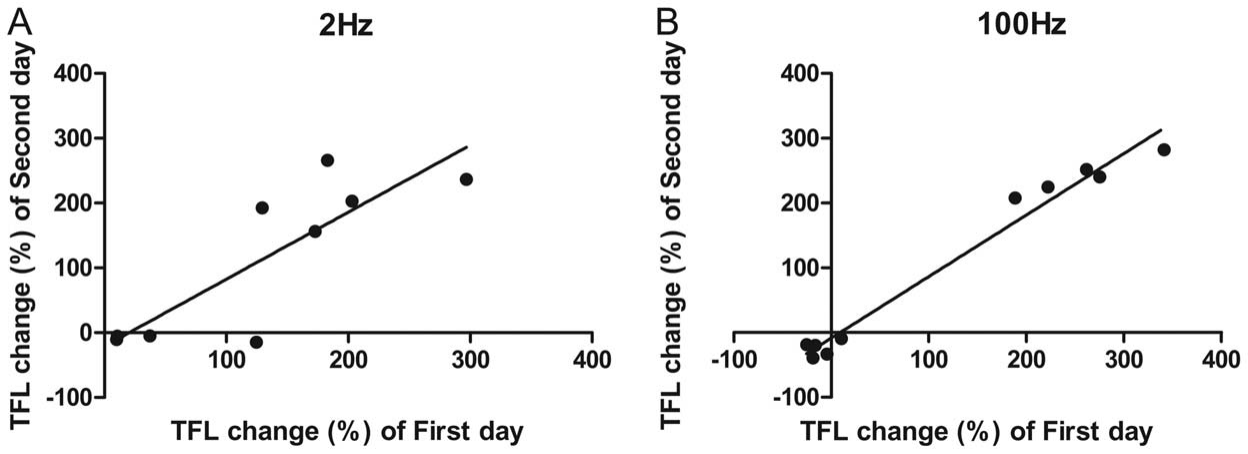
Scattergram of analgesic effect on two consecutive days of rats chosen in microarray experiment. In 2 Hz treated groups, the HR and NR rats have well reproducible in analgesic effect (R = 0.838, *P*<0.01 Pearson Correlation) on two consecutive days were chosen (A). Meanwhile, the rats in 100 Hz treated groups have well reproducible in analgesic effect (R = 0.988, *P*<0.001 Pearson Correlation) on two consecutive days were chosen (B).

### EA Stimulation

A total of 170 male rats were used in the experiment. Eighty of them were given 2 Hz EA, other 80 were given 100 Hz EA, and the rest of 10 were given restraint without EA to serve as control group to minimize the effect of restraint stress. The Zusanli (ST36) and Sanyinjiao (SP6) acupoints were chosen, which are commonly used acupoints to study acupuncture analgesic effect on diverse pain [3], [25]. EA was given to the respective groups of rats once a day in two consecutive days. EA stimulations were performed as described before [26]. In brief, stainless steel needles of 0.3 mm in diameter and 3 mm in length were bilaterally inserted in the hind legs, one at the ST36 (2 mm lateral to the anterior tubercle of tibia), and the other at the SP6 (2 mm proximal to the upper border of medial melleolus, at the posterior border of the tibia). Square-wave electric output with constant current was generated by a programmed pulse generator HANS, LH 800 (manufactured at Astronautics and Aeronautics Aviation of Peking University) and1was delivered *via* the needles for a total of 30 min. The frequency of the stimulation was set at either 2 Hz or 100 Hz. The intensity of stimulation was increased stepwise from 0.5 to 1.0 and then to 1.5 mA, with each step lasting for 10 min. EA stimulation once a day in two successive days.

### Nociceptive Testing

Nociceptive threshold was assessed by recording the TFL induced by radiant heat [27], [28]. Focused light beam (3 mm diameter) from 12.5 W projector bulb was applied to the junction between proximal 2/3 and distal 1/3 of the tail. The projector bulb was turned off as soon as the rat flicked its tail, and a digital timer measured the TFL with the accuracy of 0.1 s. The voltage of the stimulation was adjusted to 12 V and room temperature was carefully monitored at 22±1°C to minimize the possible influence of ambient temperature on TFLs during the test [29]. We used a cut-off latency of 15 s in order to avoid possible damage to the superficial tissue of the tail. The average of three successive TFL determinations (pre-EA TFL) before EA stimulation was recorded as basal latency. The TFL, ten minutes after the ending of EA stimulation, was also assessed as EA latency. The analgesic effect of EA was represented by the average percentage change of TFL (%): [(EA latency–basal latency)/basal latency] ×100%. Results were presented as mean ± SEM and were analyzed with one-way ANOVA followed by Bonferroni’s Multiple Comparison Test.

**Figure 3:**
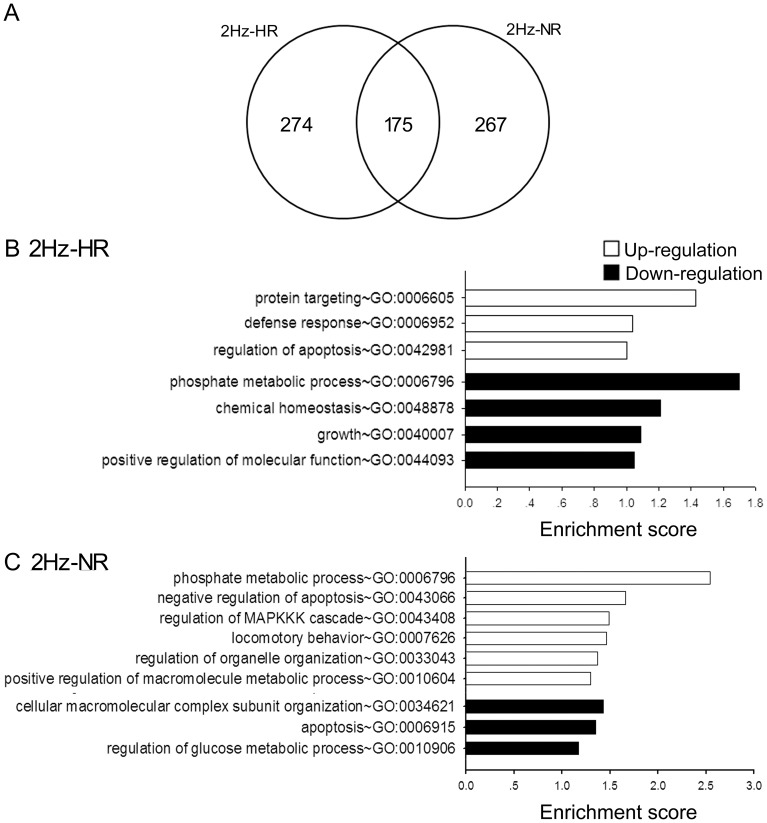
Overlapped and non-overlapped gene expression and enriched GO categories in 2 Hz-HR and NR rats. A: Venn diagram comparing the number of genes/ESTs identified as differentially expressed in HR and NR after 2 Hz EA stimulation at 1 hr time point. B and C: Enriched GO categories of specially regulated genes in 2 Hz-HR and 2 Hz-NR. The complete gene list of each GO category is accessible at: Table S3A.

### DH Tissue Harvesting

Rats were sacrificed either one hour (1 h) or 24 hours (24 h) after the last EA session on the last test day. Each rat was sacrificed by decapitation and the DH of the fifth and sixth lumbar (L5 and L6) spinal cord were quickly removed and stored immediately in cold RNAlater (Qiagen, Hilden, Germany), and then stored at −80°C till used later.

### Gene Expression Profiling and cDNA Microarray Analysis

The DH tissues from HR and NR rats sacrificed at 1 hr time point were used in microarray experiment. The cDNA microarray containing 11,444 rat genes/Expressed Sequence Tags (ESTs) was used and the microarray platform was submitted to the GEO database with the accession number GPL3498 [30], [31], [32]. The protocol of the microarray experiments and the strategy for data extraction were described as previously [30], [32]. Briefly, equal amounts of RNA samples from five restraint rat DH tissues were labeled with Cy3. The labeled RNA samples were pooled and used as reference. The RNA samples from animals following EA stimulation were individually labeled with Cy5, then Cy3-and Cy5-labeled cRNA pools were mixed to hybridize to the microarrays.

Data preprocessing was performed using Bioconductor software [33] (http://www.bioconductor.org) under the statistical programming environment R [34]. Signal intensity normalization within and between microarrays was accomplished by using intensity-dependent locally weighted scatter plot smoothing regression analysis (LOWESS). Only the genes/ESTs presenting in more than 60% samples in each group were retained for further analysis. Differentially expressed genes were then identified using the LIMMA (linear models for microarray analysis) package, and the empirical Bayes method was used to reduce the gene wise sample variances [35]. A combination of fold change ≥1.5 and a rigorous *P*-value ≤0.001 was used to identify differentially expressed genes/ESTs. The data have been submitted to GEO under accession GSE21758.

**Table 1:** The regulated genes of neuroactive ligand-receptor interaction and release of proinflammatory cytokines in 2 Hz-HR and 2 Hz-NR rats after 2 Hz EA stimulation.

*Group*	*Term*	*GenBank*	*Gene symbol*	*Log2 ratio*	*P-value*
2 Hz-HR	Neuroactive ligand-receptor interaction	NM_031349	Aplnr	0.98	0.0009316
		AF293459	Faf1	−1.06	0.000847
		L08491	Gabra2	−0.94	0.0004549
		NM_080586	Gabrg1	−1.61	5.417E-06
		NM_053296	Glrb	−1.20	0.0001422
		NM_021857	Htr1f	−1.01	2.225E-06
	Release of proinflammatory cytokines	NM_033230	Akt1	−1.22	0.0003809
		AF293459	Faf1	−1.06	0.000847
		NM_175756	Fcgr2b	1.89	1.808E-07
		NM_017090	Gucy1a3	1.82	3.023E-10
		NM_032080	Gsk3b	1.26	4.529E-06
		NM_053838	Npr2	−1.22	5.247E-05
		NM_012713	Prkcb	−1.70	2.997E-06
		NM_013012	Prkg2	−1.36	2.776E-06
		BC061979	Tsc22d3	1.17	0.0008219
2 Hz-NR	Neuroactive ligand-receptor interaction	XM_001061557	Map1b	−0.93	0.0006714
	Release of proinflammatory cytokines	NM_053619	C5ar1	1.62	0.0001809
		NM_171994	Cdc42	1.92	0.0001617
		BQ196565	Gpx2	−1.50	9.133E-05
		NM_052807	Igf1r	1.08	0.0008666
		NM_031512	Il1b	1.64	9.871E-05
		NM_012806	Mapk10	−1.92	3.064E-07
		NM_138548	Nme1	2.29	1.965E-05
		NM_175578	Rcan2	−1.27	0.0007497

Show is up-regulation (log2 ratio ≥0.585) or down-regulation (log2 ratio ≤−0.585) by ≥1.5-fold compared with control rats after EA stimulations.

### Bioinformatic Analysis

Biological themes associated with differentially expressed genes were identified by the biological process of the Gene Ontology (GO) categories using the functional annotation tool of the Database for Annotation, Visualization, and Integrated Discovery (DAVID) (http://david.abcc.ncifcrf.gov/) [36], to identify important GO categories (enrichment, EASE scores ≥1.0) and to suggest their potential biological importance. The biological process of differently expressed genes was ranked by the EASE scores based on all enriched annotation terms.

To provide a functional outline for function interpretation, regulated gene pathways were explored using Kyoto Encyclopedia of Genes and Genomes (KEGG) online database (http://www.genome.jp/kegg/). The KEGG pathways of the differentially expressed genes were also matched using the DAVID Functional Annotation Tool. DAVID gives a modified Fisher Exact *P*-Value for pathway enrichment analysis.

**Figure 4:**
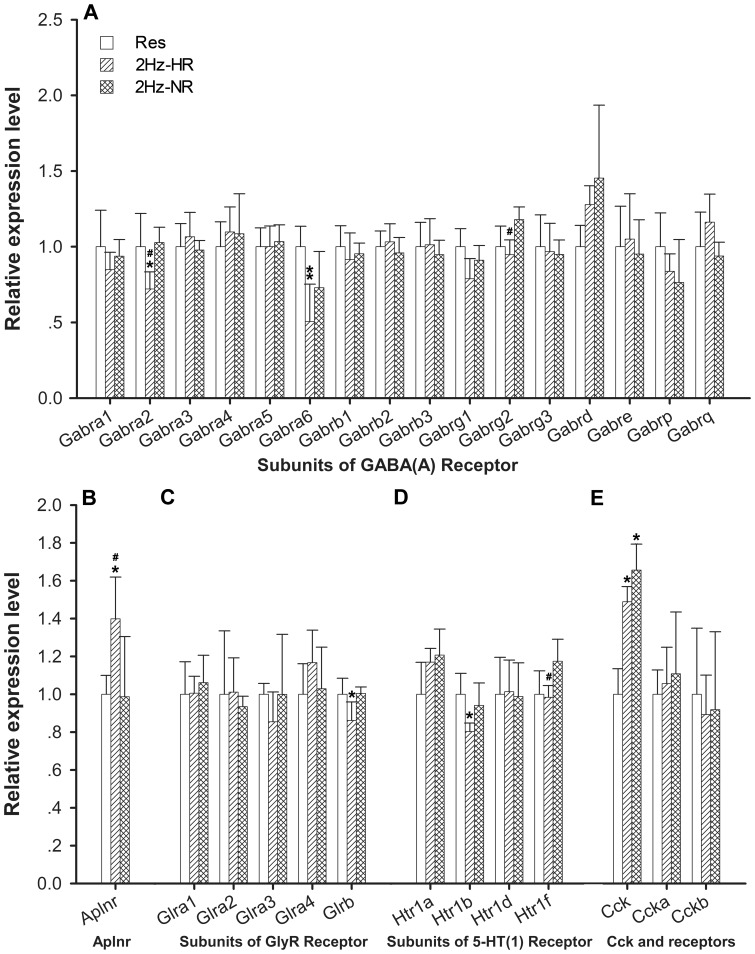
Expression of neurotransmitter receptors-related genes at 1 h. Data (mean ± SD) are normalized to restraint group. qPCR was conducted with triplicate experiments in each. One way ANOVA and Tukey’s HSD post-hoc test was used. *P<0.05, **P<0.01 *vs.* restraint group; ^#^ *P*<0.05 vs. NR group.

### Real-Time RT-PCR

RNA samples were from animals sacrificed at 1 hr and sacrificed at 24 hr time point after EA stimulation. Aliquots of the RNA samples were used in Real-time RT-PCR (qPCR). Two micrograms of each total RNA sample were used for cDNA synthesis using iScript™ cDNA Synthesis Kit (Bio-Rad, Hercules, CA). cDNA samples were placed on ice and stored at −20°C until further use. Prior to the analysis, 20 µl of each cDNA sample was diluted with 180 µl of MilliQ water. qPCR reactions were performed with Prism 7900 Sequence Detection System (Applied Biosystems, Foster City, CA). For each reaction, 1 µl of each diluted cDNA sample was added to a mixture containing 12.5 µl of 2× SYBR green II qRT-PCR kit (Toyobo, Osaka, Japan), 1 µl of each primer (5 µM), and 10.5 µl of MilliQ water. Cycling conditions were 10 min 95°C, followed by 40 cycles of 15 s at 95°C and 1 min at 60°C. After cycling, a melting protocol was performed with 15 s at 95°C, 1 min at 60°C, and 15 s at 95°C, to control product specificity. The fold change (FC) of target gene cDNA relative to glyceraldehyde-3-phosphate dehydrogenase (*Gapdh*) endogenous control was determined as follows: FC = 2^−ΔΔCt^, where ΔΔCt = (Ct_Target_–Ct_Gapdh_)test–(Ct_Target_–Ct_Gapdh_)control. Ct values were defined as the number of the PCR cycles at which the fluorescence signals were detected. The primer sequences are listed in Table S1. Data are presented as mean ± SEM and analyzed with one-way ANOVA followed by Tukey’s HSD post-hoc test.

**Figure 5:**
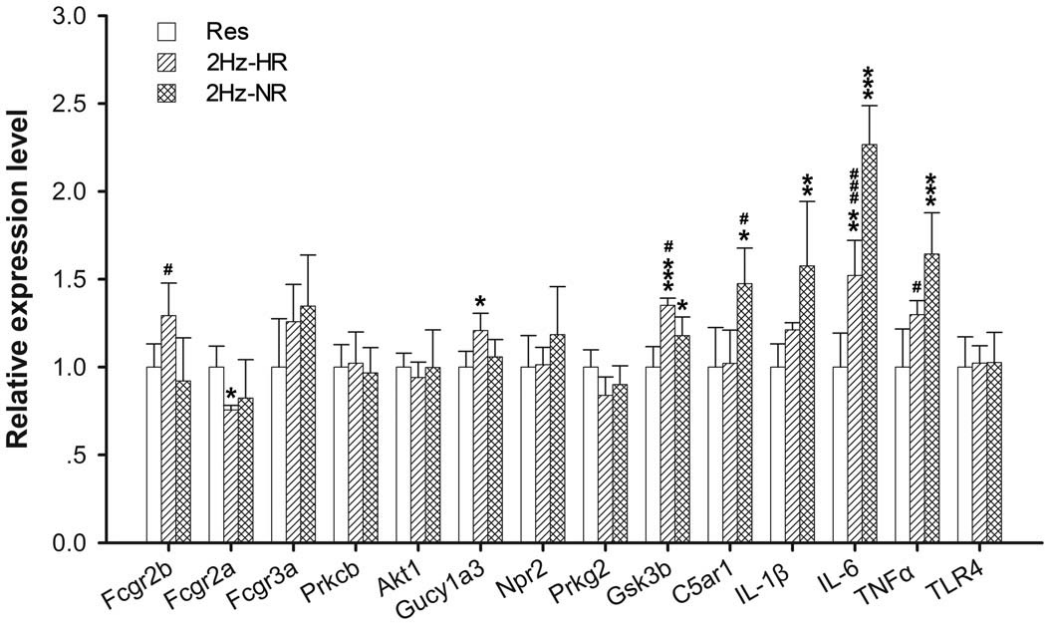
Expression of release of proinflammatory cytokines-related genes at 1 h. Data (mean ± SD) are normalized to restraint group. qPCR was conducted with triplicate experiments in each. One way ANOVA and Tukey’s HSD post-hoc test was used. *P<0.05, **P<0.01, ***P<0.001 *vs.* restraint group; ^#^ *P*<0.05, ^###^ *P*<0.001 vs. NR group.

## Results

### High-responders and Non-responders were Defined Based on Distinct Analgesic Effects Induced by EA at Different Frequencies

Analgesic effects induced by 2 Hz and 100 Hz EA were estimated by the TFL test in rats and the average percentage change in TFL induced by EA stimulations once a day in two consecutive days. The distribution of sensitivity towards EA analgesia in the testing population indicated that majority of the rats (Fig. 1A. B), no matter tested in the first day or the second day, showed some sensitivity towards 2 Hz or 100 Hz EA analgesia (150≥ TFL changes >0) (responder, R). However, a small portion of them fell in either high-sensitivity (HR; TFL changes >150) or low-sensitivity (NR; TFL changes ≤0) category. There was no significant difference in the basic TFL among different treatment groups. In addition, restriction did not appear to affect their TFL (data not shown). Among the 80 rats received 2 Hz EA on the second day, 54 rats showed modest analgesic effects (mean TFL increase ratio of 65.08%) and were classified as R. Nineteen rats showed high-sensitivity with the mean TFL increased 209.15% and were classified as HR. In contrast, 7 rats were NR (mean TFL decreased 5.69%) (Fig. 1C). To the 80 rats given 100 Hz EA, 49, 8 and 23 rats fell into R (mean TFL increased 48.09%), HR (mean TFL increased 234.74%) and NR (mean TFL increased 22.04%), respectively (Fig. 1 D).

**Figure 6:**
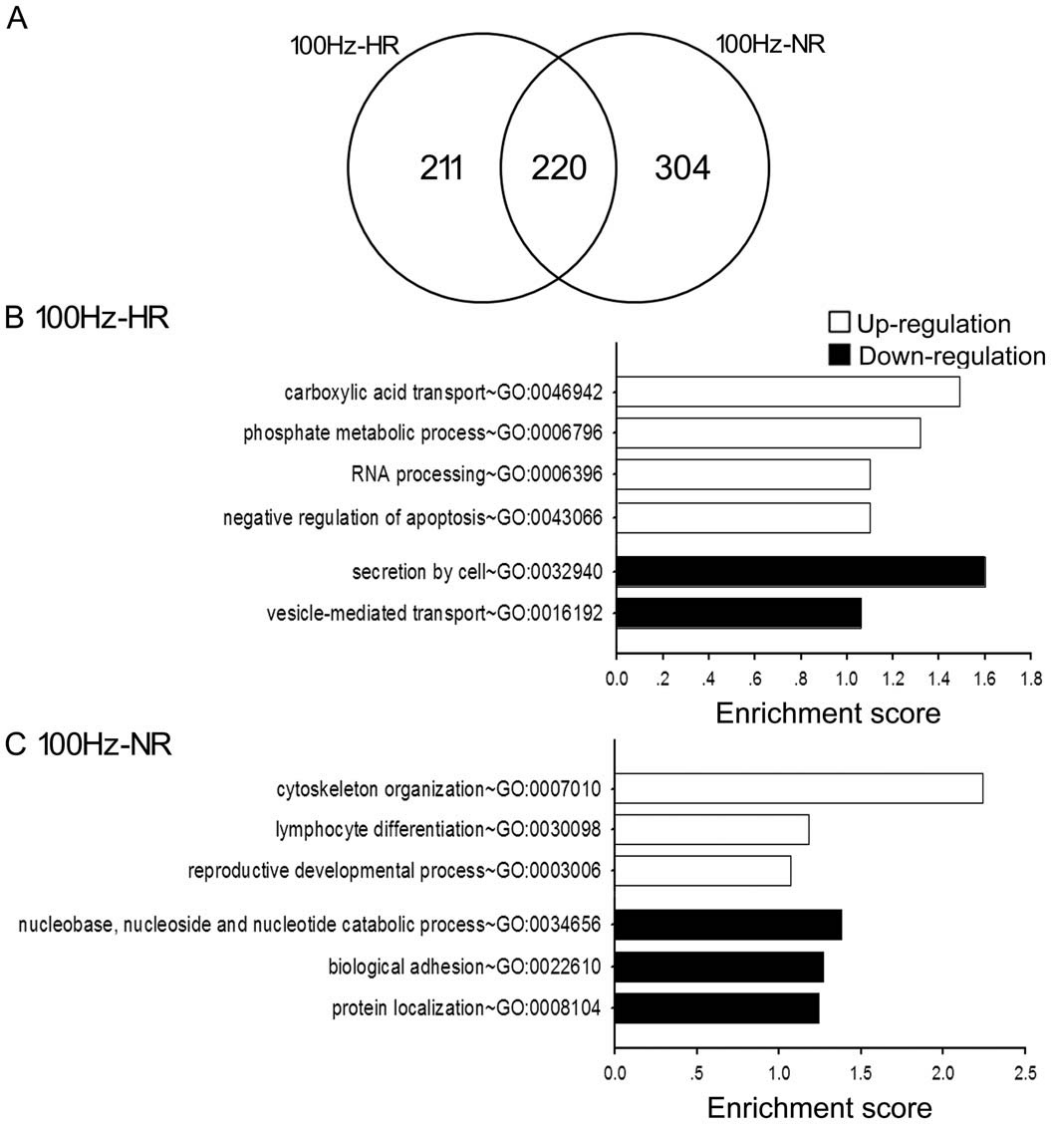
Overlapped and non-overlapped gene expression and enriched GO categories in 100 Hz-HR and NR rats. A: Venn diagram comparing the number of genes/ESTs identified as differentially expressed in HR and NR after 100 Hz EA stimulation at 1 hr time point. B and C: Enriched GO categories of specially regulated genes in 100 Hz-HR and 100 Hz-NR. The complete gene list of each GO category is accessible at Table S5A.

### Transcriptome Modulations were Detected in the HR and NR Individuals

According to the analgesic response to EA treatment in the second day, 26 rats (19 HR *vs.* 7 NR) in 2 Hz EA and 31 rats (8 HR *vs.* 23 NR) in 100 Hz EA were chosen for further analysis. Among these animals, individuals that presented best reproducibility in the two assessments were selected for microarray experiment based on statistical analysis of two-tailed Pearson’s Correlation and paired student’s *t*-test. Finally, nine rats (5 HR *vs.* 4 NR; R = 0.838, *P*<0.01; *t* = 0.75, df = 8, *P* = 0.474) in 2 Hz EA treatment group and 10 rats (5 HR *vs.* 5 NR; R = 0.988, *P*<0.001; *t* = 2.04, df = 9, *P* = 0.072) in 100 Hz EA treatment group were sacrificed at 1 hr time point after the last EA stimulation and the tissues of DH region were used to be processed for microarray experiment (Fig. 2). After filtering for high-quality array data (*See Methods*), the global transcriptional profiling of the DH region with 8442, 8382, 9498, and 9735 genes/ESTs in the 2 Hz-HR, 2 Hz-NR, 100 Hz-HR, and 100 Hz-NR groups was investigated, respectively. Using the high stringent analysis with Limma at the combination of fold change ≥1.5 and a rigorous *P*-value ≤0.001, significant differences in gene expression were observed after the EA stimulations compared with the restraint-control group. There were 449, 442, 431, 524 genes/ESTs were identified to be significantly changed level of expression in the 2 Hz-HR, 2 Hz-NR, 100 Hz-HR, and 100 Hz-NR group, respectively.

**Figure 7:**
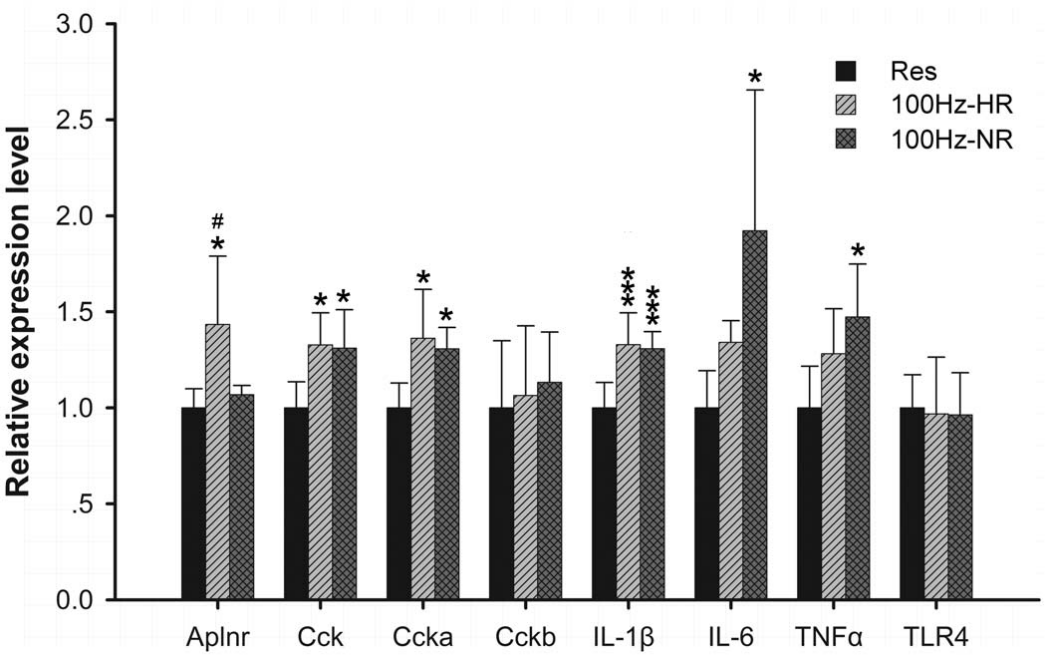
Gene expression detected in 100 Hz-HR and NR at 1 h by qPCR. Data (mean ± SD) are normalized to restraint group. qPCR was conducted with triplicate experiments in each. One way ANOVA and Tukey’s HSD post-hoc test was used. *P<0.05, **P<0.01 *vs.* restraint group; ^#^ *P*<0.05 vs. NR group.

### Different Gene Expression Response to 2 Hz EA between HR and NR

In the animals treated with 2 Hz EA, 175 genes/ESTs were similarly co-regulated in HR and NR. The changes in expression level were in the same direction in HR and NR (Fig. 3A, Table S2A). Besides, the expression of 274 and 267 genes/ESTs was specifically regulated in HR and NR groups, respectively (Fig. 3A; Table S2B, S2C). These differentially regulated genes were subjected to DAVID analysis to identify their potential biological themes. Genes enriched in 16 GO categories belong to the ontology of “biological process” (EASE score ≥1) (Fig. 3B, 3C; Table S3A). Among these enriched GO categories, 13 of them were mainly related with cell cycle process, which did not have direct link to the effect of EA analgesia and pain modulation [4], [21], [37], [38]. Interestingly, the immune function related term defense response (GO:0006952) was enriched in 2 Hz-HR specific genes and the term regulation of MAPKKK cascade (GO:0043408) was enriched in 2 Hz-NR specific genes. With KEGG pathway analysis, B cell receptor signalling pathway (rno04662) was enriched in the 2 Hz-HR genes (*P*-Value ≤0.05) and three pathways (Ribosome (rno03010), Focal adhesion (rno04510) and Oocyte meiosis (rno04114)) were enriched in the 2 Hz-NR genes (Table S3B).

According to the hint that the immune function by the GO categories and KEGG pathways analysis as mentioned above and the neurochemical mechanisms implicated in pain and analgesia by literature exploration, we mapped the specially regulated genes identified in two extreme phenotype groups (HR and NR) according to these two functional characteristics. We thus identified that neuroactive ligand-receptor interaction and release of proinflammatory cytokines may play significant roles in individual variations in nociception response to 2 Hz EA (Table 1).

**Figure 8:**
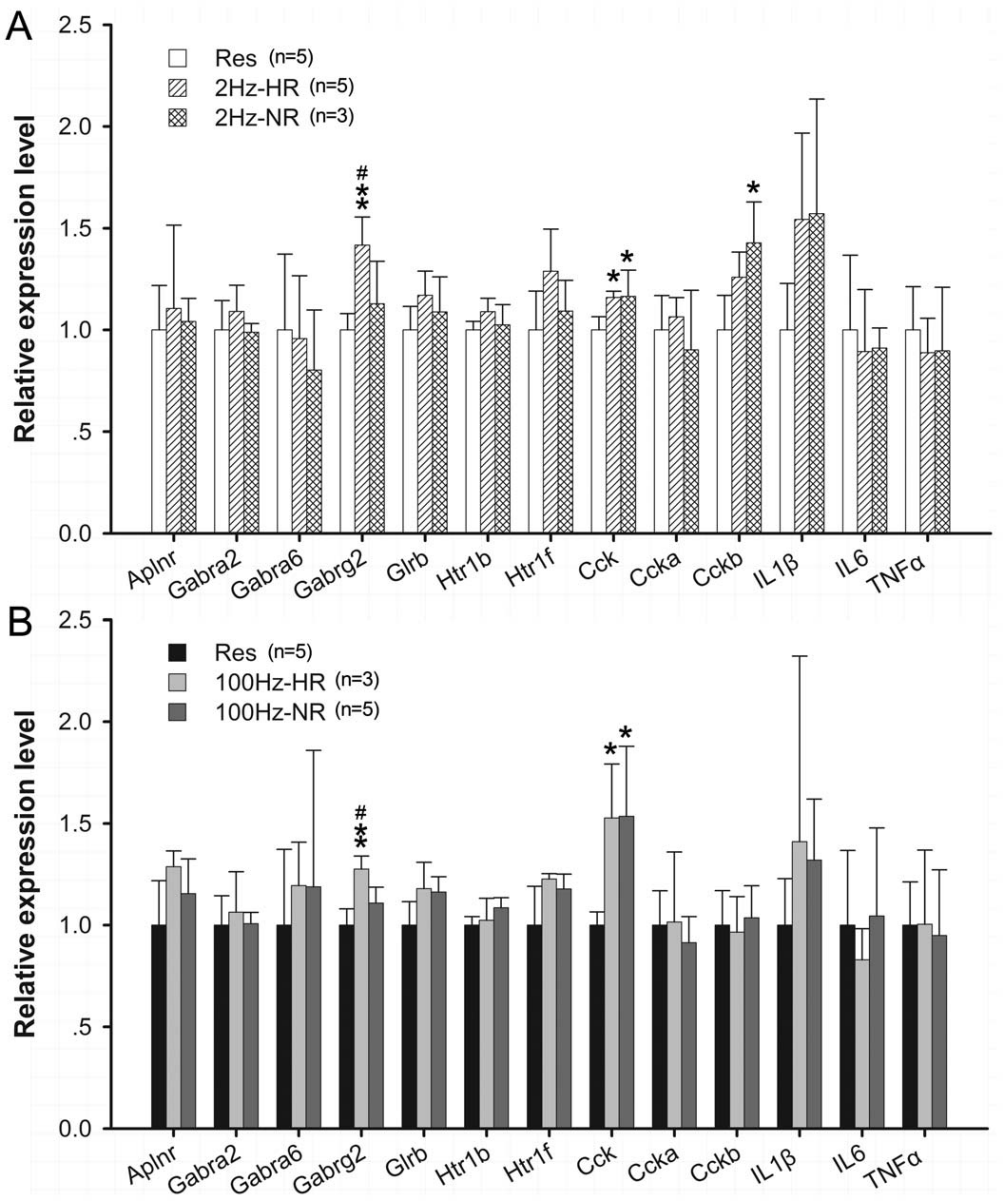
Gene expression at 24 hr time point after EA stimulation. Data (mean ± SD) are normalized to restraint group. qPCR was conducted with triplicate experiments in each. One way ANOVA and Tukey’s HSD post-hoc test was used.

With respect to the neuroactive ligand-receptor interaction, *Aplnr* was up-regulated and *Gabra2*, *Gabrg1*, *Htr1f*, and *Glrb* were down-regulated in the 2 Hz-HR group. In contrast, there were no direct regulated genes related to neuroactive ligands or receptors in 2 Hz-NR group. With respect to the release of proinflammatory cytokines, mostly regulated genes implicated a trend of inhibiting the release of proinflammatory cytokines in 2 Hz-HR group, while in the 2 Hz-NR group, the regulated genes implicated a trend of inducing the release of proinflammatory cytokines.

### Regulation of Neurotransmitter Receptors-related Gene Expression in 2 Hz-HR and 2 Hz-NR rats

Based on the results of the microarray analysis and suggestions regarding the neuroactive ligand–receptor interaction, qPCR was employed for detailed analysis of differential regulation of neurotransmitter receptors in 2 Hz-HR and 2 Hz-NR rats. The genes selected for this analysis included *Aplnr* and all subunits of GABA_A_ receptor (*Gabra1–6*, *Gabrb1–3*, *Gabrg1–3*, *Gabrd*, *Gabre*, *Gabrp*, and *Gabrq*), glycine receptor (*Glra1–4* and *Glrb*), and 5-HT1 receptor (*Htr1a*, *Htr1b*, *Htr1d*, and *Htr1f*). In 2 Hz-HR group, the mRNA level of *Aplnr* was up-regulated and the mRNA levels of *Gabra2*, *Gabra6*, *Gabrg2*, *Glrb*, and *Htr1b* were down-regulated compared with the control group (Fig. 4A–4D). However, there was no significant difference in the expression of these genes in the 2 Hz-NR group comparing with that in the control group. Furthermore, the mRNA levels of *Aplnr*, *Gabra2*, *Gabrg2*, and *Htr1f* were significantly different between 2 Hz-HR and 2 Hz-NR groups (Fig. 4A–4D).

In addition, Cck is widely distributed in various brain areas and the spinal cord and exerts many physiological functions. Previous studies have clearly shown both Cck release and the density of Cck receptors are closely associated with individual sensitivity to EA [4]. NR rats had a remarkable increase in Cck release [16]. Meanwhile, the expression of *Cck-a* receptor at mRNA level was significantly higher in the rat hypothalamus of NR than HR following low-frequency EA [9]. Cck receptors (*Cck-a* and*-b*) mRNA in the hypothalamus were also increased by high-frequency EA in NR rats [10]. To assess whether the differences in expression pattern between HR and NR rats in the brain also present in DH region, the mRNA levels of *Cck*, *Cck-a* and *Cck-b* were examined using qPCR. As seen in Figure 4E, the expressions of *Cck* and its receptors (*Cck-a* and *Cck-b*) in the DH were not significantly different between HR and NR after 2 Hz EA stimulation. Compared with control group, the expression of *Cck* was significantly increased in both HR and NR in the DH after 2 Hz EA stimulation (Fig. 4E).

**Figure 9:**
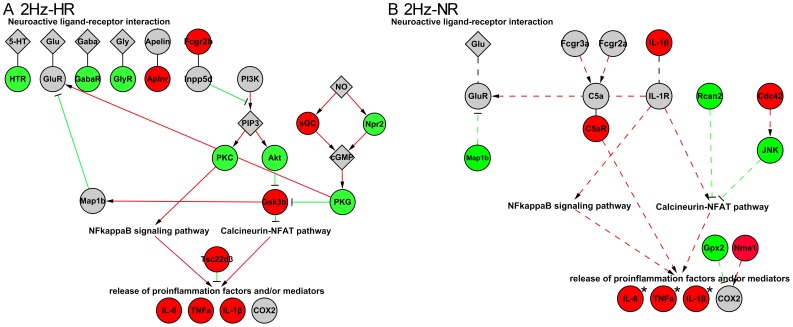
Ideogram illustration depicting the different regulated genes in neural-immune system in HR and NR after 2 Hz EA. Regulated gene network in the DH at 1 hr time point after 2 Hz EA of 2 Hz-HR group (A) and 2 Hz-NR group (B). The expressions of neurotransmitter receptors were regulated in HR, not in NR. Regulated genes related with the release of proinflammatory cytokines were also shown. The mRNA levels of *IL-1β*, *IL-6*, and *TNFα* were increased in both HR and NR, but higher increased in NR compared with HR. Edges (lines) connecting nodes (genes) represent regulatory interactions such as inhibits (T shape) or activates (Arrow shape). Red node indicates gene up-regulation. Conversely, blue node indicates down-regulation, grey node was non-regulation.

### Regulation of Proinflammatory Cytokines-related Gene Expression in 2 Hz-HR and 2 Hz-NR Rats

The expression pattern of genes related to the release of proinflammatory cytokines was found to be different between the 2 Hz-HR and 2 Hz-NR groups. Compared with control group, the expression of *Fcgr2a* was down-regulated and the *Gucy1a3* was up-regulated in the HR group, while the expression of *C5ar1* was up-regulated in the NR group after 2 Hz EA stimulation by qPCR analysis (Fig. 5). *IL-1β*, *IL-6* and *TNFα*, three important proinflammatory cytokines, increased their expression in both HR and NR after 2 Hz EA stimulation (Fig. 5). Interestingly, the mRNA levels of *Fcgr2b* and *Gsk3b*, which could depress the proinflammatory cytokines release, were significantly increased in 2 Hz-HR group than 2 Hz-NR group (Fig. 5). Meanwhile, the expression of *C5ar1*, which could facilitate the proinflammatory cytokines release, was significantly increased in 2 Hz-NR group than 2 Hz-HR group (Fig. 5). Furthermore, higher levels of *IL-1β*, *IL-6* and *TNFα* mRNA were observed in 2 Hz-NR group compared to 2 Hz-HR group (Fig. 5). However, toll-like receptor 4 (TLR4) mRNA levels, an index of glial activation [39], had no difference between HR and NR after 2 Hz EA (Fig. 5).

### Different Gene Expression Response to 100 Hz EA between HR and NR

With 100 Hz EA treatment, 211 and 304 genes/ESTs were identified to have specific regulations in HR and NR group, respectively (Fig. 6A; Table S4). Based on GO annotation, six GO categories were enriched in 100 Hz-HR specifically regulated genes and six in 100 Hz-NR specifically regulated genes (Fig. 6B, 6C; Table S5A). Importantly, GO terms of secretion by cell (GO:0032940) and vesicle-mediated transport (GO: 0016192) were enriched in the 100 Hz-HR genes, the term lymphocyte differentiation (GO:0030098) was enriched in the 100 Hz-NR group. In KEGG pathway analysis, Ribosome (rno03010) was enriched in the 100 Hz-HR group while two pathways of Ribosome (rno03010) and Fc gamma R-mediated phagocytosis (rno04666) were enriched in the 100 Hz-NR genes (Table S5B).

Unlikely multiple genes with their expression pattern significantly different between HR and NR noticed in the 2 Hz EA study, we did not see striking differences in expression pattern in genes potentially related to variations in analgesic response to 100 Hz EA. The mRNA levels of neurotransmitter receptors and proinflammatory cytokines were also detected in 100 Hz EA groups by qPCR. In neurotransmitter receptors, only the expression of *Aplnr* was significantly increased in HR rats after 100 Hz EA stimulation compared with NR group and control group (Fig.****7). The expression of *Cck* and *Cck-a* were increased in both HR and NR rats after 100 Hz EA stimulation, and there was no difference in expression pattern between HR and NR rats (Fig. 7). In respect of proinflammatory cytokines, the expression of *IL-1β* was up-regulated in both HR and NR, while *IL-6* and *TNFa* mRNA were only significantly increased in NR group after 100 Hz EA stimulation (Fig. 7). However, the mRNA level of *TLR4* was similar in HR and NR (Fig. 7).

### Time Effects of EA Stimulation

In order to determine whether the gene regulation response after EA stimulation observed at 1 hr time point would sustain for a longer period of time, the gene expression activity was also examined at 24 hr time point after EA stimulation by qPCR. As shown in Figure 8, approximately 90% of the genes the expression of which has been altered at 1 hr time point returned to baseline at 24 hr time point compared the control group. However, the expression of *Cck* was still up regulated at at 24 hr time point in both HR and NR after 2 Hz and 100 Hz EA (Fig. 8). The expression of *Cck-b* gene was significant increased in 2 Hz-NR group only when compared with the control group at 24 hr time point (Fig. 8). The *Gabrg2* mRNA levels was significantly increased only in HR group following 2 Hz and 100 Hz stimulation but not in NR or control groups at 24 hr time point (Fig. 8).

## Discussion

In the present study, both 2 Hz and 100 Hz EA could produce a good analgesic efficacy in most rats. On the other hand, the analgesic effects of EA on rats showed marked individual variations (Fig. 1), although there was no significant difference in basal nociceptive threshold of HR, R, and NR rats, as measured by TFL test. It is well known that the analgesic effect of EA can be produced by transcriptional and non-transcriptional mechanisms in the nervous system. Therefore, cDNA microarray technique was used to examine the gene expression response in the DH region in order to understand the underlying mechanisms why there are individual differences in EA analgesia.

The gene expression response was profiled at 1 hr time point after EA stimulation. The time point was chosen due to the fact that the antinociceptive effects of EA were thought to be a short term response in normal animals. The gene expression profiles of DH were compared between HR and NR rats, which were classified based on their analgesic response to EA analgesia. HR and NR rats with reproducible analgesic response to EA analgesia in two consecutive days (Fig. 2), were used in the microarray experiment in order to minimize the random environmental factors. As shown in this study, HR rats and NR rats exhibited significant differences in gene expression response to 2 Hz or 100 Hz EA stimulations.

### Potential Mechanisms of Individual Variations in Response to 2 Hz EA Analgesia

Compared with the gene expression profiles in response to 2 Hz EA, the genes related with neuroactive ligand-receptor interaction were more obviously regulated in the HR *vs.* NR rats and the genes related with release of proinflammatory cytokines were more dramatically up-regulated in NR (Fig. 9). For neuroactive ligand-receptor interaction, the mRNA expressions of some neurotransmitter receptors, involved in Aplnr receptor and GABA_A_ receptor, glycine receptor, and 5-HT1 receptor, were regulated in HR rats. However, these genes were not significantly regulated in NR rats. Consistent with the microarray analysis, qPCR further demonstrated that the expression of *Aplnr* was up-regulated and the expressions of *Gabra2*, *Gabra6*, *Gabrg2*, *Glrb*, and *Htr1b* were down-regulated in the HR rats’ DH region response to 2 Hz EA (Fig. 4). Meanwhile, these genes were not significantly regulated in the NR rats’ region after 2 Hz EA. Furthermore, the mRNA levels of *Aplnr*, *Gabra2*, *Gabrg2*, and *Htr1f* were significantly different between HR and NR groups (Fig. 4). Previous studies confirmed that low frequency EA was effective in treatment of neuropathic pain through mediating the neurotransmitters of GABA, 5-HT or glycine as well as their corresponding receptors [40], [41]. Note worthily, the expression of *Aplnr*, a G protein-coupled receptor, was up-regulated in HR rats. The endogenous ligand for Aplnr was apelin, and the apelin-Aplnr system is widely distributed in both CNS and periphery, which inhibit the adenylate cyclase activity [42], [43], [44]. One study showed that intra-cerebroventricular administration of apelin could produce a dose-and time-dependent antinociceptive effect by acting on Aplnr and μ-opioid receptor [45]. Therefore, this study implicated that the apelin-Aplnr system would be a new candidate system that might participate in EA analgesia and was related with individual differences to 2 Hz EA stimulate. On the other hand, the expression of *Cck* was significantly increased after 2 Hz EA stimulation and not different between HR and NR in DH. This result that *Cck* mRNA level was increased after EA and there was no difference between HR and NR was consistent with the previous studies in DH and supraspinal regions [6], [10], [46], while the Cck protein level was higher in NR than HR in the midbrain periaqueductal gray and the perfusate of the rat spinal cord [6], [16]. The mRNA level of the *Cck-a* receptor in DH had no significant difference in NR and HR of this study, which was inconsistent with one previous study in hypothalamus that *Cck-a* mRNA level was high expressed in NR than HR [8]. This discrepancy may be due to the differences in the examined CNS regions (DH *vs.* hypothalamus) and differences in the EA stimulation conditions used. Generally speaking, these different regulations in neurotransmitter receptors’ genes in HR and NR suggested that neurotransmitter system could be mainly active in HR rats, but not in NR rats.

With respect to the release of proinflammatory cytokines, the regulated genes in HR rats mainly inhibited release of proinflammatory cytokines after EA stimulation. However, in NR rats the response genes would induce the release of proinflammatory cytokines. For example, the expression of *Fcgr2b*, *GSK3b* and *Tsc22d3* were up-regulated in HR rats, which could significantly inhibit the release of the proinflammatory cytokine [47], [48], [49]. The expression of *IL-1β* was up-regulated in NR rats. IL-1β is a proinflammatory cytokine, which not only induces the release of other proinflammatories but also plays a major role in nociceptive modulation in the CNS and can be nociceptive and produce hyperalgesia [50], [51], [52]. *IL-6* and *TNFα* mRNA expression, two other important proinflammatory cytokines, also significantly increased their expression in NR than HR after 2 Hz EA stimulation (Fig. 5). It was shown that spinal proinflammatory cytokines could be induced by multiple opioid administrate in opposing both acute and chronic opioid analgesia in normal animals [53]. This study implicated that the individuals had different reactivity in proinflammatory cytokines release, which resulted in different response with 2 Hz EA stimulation. Proinflammatory cytokines are key elements in the induction and maintenance of pain and are predominantly secreted by microglia, and some astrocytes in the CNS [51], [54]. However, the expression of *TLR4*, one of glial activation marker, had no significant difference between HR and NR (Fig. 5). Therefore, it is uncertain whether the different expressions of proinflammatory cytokines between HR group and NR group were due to the different activity of glia in 2 Hz EA analgesia.

### Differential Alterations of Gene Expression in HR and NR rats After 100 Hz EA Analgesia

In 100 Hz EA, HR and NR rats also exhibit differential expression response to 100 Hz analgesia in the DH. The GO enriched analysis found that category ‘vesicle-mediated transport’ (GO: 0016192) related to neural function was enriched with down-regulated genes in HR rats and ‘lymphocyte differentiation’ (GO:0030098) related to immune function was enriched with up-regulated genes in NR rats (Fig. 6). These two enriched GO categories in respective HR and NR were some similar to the findings that different regulation of neural-immune related genes in response to 2 Hz EA. Unconformity with the results of 2 Hz EA, there was no significant gene expression difference in subunits of GABA_A_ receptor, 5-HT1 receptor or GlyR receptor between HR and NR after 100 Hz EA. However, the expression of *Aplnr* was also increased in HR rats compared with NR and the control rats, further indicating that the apelin-Aplnr system would involve in 2 Hz/100 Hz EA analgesia. In proinflammatory cytokines, although the gene expression of *IL-1β* did not differ, *IL-6* and *TNFα* were more increased in NR compared with HR response to 100 Hz EA. This result suggested that different gene expression response of spinal proinflammatory cytokines were also related with individual variations in 100 EA analgesia, which the gene of spinal proinflammatory cytokines were higher up-regulated in NR than HR.

### Time Effects of EA Stimulation

Twenty-four hours after EA stimulation, major portion of the regulated genes had return to baseline compared with the control group (Fig. 8). This result suggested that the regulation of gene expression in naïve rat response to EA stimulations had time-dependent changes and the burst of gene expression changes were regulated at early stage. This time-dependent changes in gene expression response to EA stimulation were in accordance with the duration of EA analgesia effect on the physiological stat in previously studies [2], [55].

It is important to uncover the mechanisms about why distinct classes of individuals differ in response to EA analgesia, which could have considerable clinical value for the practice of acupuncture and the treatment of pain. This study provides a systematic view of gene expression variations in spinal DH region with individual response to 2 Hz or 100 Hz EA analgesia. The gene expression data generated in this study can serve as a future resource to elucidate the genetic underpinnings of individual variations in response to EA analgesia. Furthermore, genes of certain neurotransmitter receptors were more prominent regulated in the HR, and proinflammatory cytokine related genes were notably up-regulated in NR compared with HR after EA stimulation. Our finding suggested that different responsiveness of neural-immune system in response to EA stimulation could have implications for elucidating the basis of individual differences in EA analgesia.

## Supporting Information

Table S1 **Sequences of primers for qRT-PCR.**
(XLS)

Table S2 **Co-regulated (A) and special-regulated genes (B, C) lists in HR and NR rats by 2 Hz EA stimulation.**
(XLS)

Table S3 **The genes lists of enriched GO categories (A) and KEGG pathways (B) in 2Hz-HR and 2Hz-NR rats.**
(XLS)

Table S4 **Co-regulated (A) and special-regulated genes (B, C) lists in HR and NR rats by 100 Hz EA stimulation.**
(XLS)

Table S5 **The genes lists of enriched GO categories (A) and KEGG pathways (B) in 100Hz-HR and 100Hz-NR rats.**
(XLS)
